# EHMTI-0244. Optogenetic elicitation of cortical spreading depression in unanesthetized, head-restrained mice

**DOI:** 10.1186/1129-2377-15-S1-F3

**Published:** 2014-09-18

**Authors:** SM Baca, A Barth, I Mody, A Charles

**Affiliations:** 1 Neurology Dept., University of California Los Angeles, Los Angeles, USA

## Introduction

We tested the hypothesis that cortical spreading depression (CSD) could be initiated by optogenetically activating astrocytes in unanesthetized mice. Previously, we demonstrated that depolarizing astrocytes expressing the light-responsive channelrhodopsin-2 receptor (ChR2) elicited CSD in brain slices and in anesthetized mice.

## Aims

Our principal aim was to demonstrate that CSD elicited optogenetically was not due to slice conditions, nor dependent on anesthetic agents.

## Methods

In mice, the ChR2 receptor was selectively expressed in astrocytes expressing glial fibrillary acidic protein (GFAP+). Mice were anesthetized to expose the skull and burr holes were made for local field potential (LFP) recordings in homotypical cortical regions. A custom stereotaxic frame immobilized the head and following recovery the mice are free to move on a treadmill (i.e., floating foam sphere). A blue laser was used to activate Chr2-expressing astrocytes through the skull and while recording LFPs.

## Results

Consistent with our previous work in slice and in anesthetized mice, blue light activation of ChR2-expressing astrocytes elicited CSDs. Multiple CSDs could be elicited within a single animal at the same location although stimulus duration varied. Green light of equal intensity failed to produce CSDs. Local LFPs from the stimulated hemisphere exhibited large DC changes that define CSD as well as high frequency discharges prior to the large DC deflection.

## Conclusions

Optogenetic approaches to the study of CSD in awake, moving animals is possible. Depolarizing populations of astrocytes is sufficient to elicit CSD and suggest that astrocyte dysfunction can play an instigating role in CSD generation.

No conflict of interest.

